# Supplementary material for: Content-rich biological network constructed by mining PubMed abstracts
Source: BMC Bioinformatics. 2004 Oct 8;5:147. doi: 10.1186/1471-2105-5-147 (PMC528731; doi:10.1186/1471-2105-5-147)
Supplement: Additional File 2 — The original results of the above study (non-essential files are deleted to keep the file size under the limit set by BMC bioinformatics). [file 1471-2105-5-147-S2.bz2 › chilibotAdditionalFile2/dip10/249ID7760852E1003/html/RARA_RXRA.html]

 


 **RARA** and **RXRA** 
  
Found 92 abstracts in PubMed, retrieved 10.  
 

 What does Google say? 
 PDF only 
| .edu only 

---

**Interactive relationship** (e.g. stimulation, inhibition, etc)

**Neutral relationship**- The recent discovery of the interaction between nuclear IGFBP 3 and 9 cis retinoic acid receptor alpha  [ **RARA** ]  retinoid X receptor alpha  [ **RXRA** ]  RXRalpha, a nuclear receptor, and its involvement in the regulation of transcriptional signaling and apoptosis represents an important paradigm shift in the understanding of IGFBP function.  Ref: 12379488 J Endocrinol, 2002

**Non-interactive relationship** (e.g. studied together, co-existance, homology, etc.)

- CONCLUSIONS Bexarotene treatment at clinically relevant concentrations causes apoptosis of CTCL cell lines in association with activation of caspase 3 and cleavage of poly ADP Ribose polymerase, as well as down regulation of retinoid X receptor alpha  [ **RXRA** ] , retinoic acid receptor alpha  [ **RARA** ] , and survivin.  Ref: 12006543 Clin Cancer Res, 2002
- PP1c specifically interacts with VDR but not retinoic acid receptor alpha  [ **RARA** ]  and retinoid X receptor alpha  [ **RXRA** ]  in yeast.  Ref: 12036952 J Biol Chem, 2002
- Bexarotene treatment suppressed the expression of retinoid X receptor alpha  [ **RXRA** ]  and retinoic acid receptor alpha  [ **RARA** ]  proteins in all three lines compared with untreated controls.  Ref: 12006543 Clin Cancer Res, 2002
- Reverse transcription polymerase chain reaction analysis showed that the retinoic acid receptors RAR alpha  [ **RARA** ] , RAR  [ **RARA** ]  gamma, and retinoid X receptor alpha  [ **RXRA** ]  were present in the cells.  Ref: 11678760 BJU Int, 2001
- Sequence comparisons and phylogenetic analyses reveal that these ascidian nuclear receptors are representative of five of the six previously defined nuclear receptor subfamilies and are apparent homologues of retinoic acid NR1B  [ **RARA** ] , retinoid X NR2B  [ **RXRA** ] , peroxisome proliferator activated NR1C, estrogen related NR3B, neuron derived orphan NOR NR4A3, nuclear orphan NR4A, TR2 orphan NR2C1 and COUP orphan NR2F3 receptors.  Ref: 12141458 Int J Dev Biol, 2002
